# The Genus *Thionia* Stål, 1859 (Hemiptera: Auchenorrhyncha: Issidae) in Colombia: Highlighting the Value of Entomological Collections

**DOI:** 10.1007/s13744-022-01002-4

**Published:** 2022-11-18

**Authors:** Camilo Andrés Llano-Arias, Giovany Guevara, Charles R. Bartlett

**Affiliations:** 1 Grupo de Investigación BioNat, Facultad de Ciencias Exactas y Naturales, Universidad de Caldas, Manizales, Colombia; 2 Grupo de Investigación en Zoología (GIZ), Departamento de Biología, Facultad de Ciencias, Universidad del Tolima, Ibagué, Colombia; 3 Associate Professor of Entomology, Department of Entomology and Wildlife Ecology, University of Delaware, Newark, Delaware USA

**Keywords:** Biological collections, Fulgoroidea, True hoppers, Planthoppers, Life zones, Natural history collections

## Abstract

Entomological collections represent a key source of information about the biological heritage of a country. However, the taxonomy and knowledge of many arthropods from megadiverse countries are underrepresented in these natural history collections as is the case with several planthoppers of the suborder Auchenorrhyncha. Issidae are fulgoromorphs distributed worldwide, except the poles and Greenland. Despite this ubiquity, Colombian planthoppers remain very poorly known and studied. Our objective was to provide the first consolidated records and distributional data for Colombian Issidae. We used reports of the representative genus *Thionia* deposited in biological collections in Colombia. In addition, we linked voucher specimen information and Olson’s life zones showing an inter-Andean valley and Eastern Andean Cordillera distribution within Colombia. Our survey of Colombian biological collections revealed 55 individuals of the genus *Thionia* Stål, 1859 (53 adults, 2 immatures [nymphs]), which were collected by different methods; however, many of those records may be opportunistic. This genus (and its species) needs further study, with systematic and ecological revision, as is the case with other terrestrial Colombian hemipterans. Our consolidated records represent an advance to the knowledge of Issidae (and *Thionia* specifically) for the Neotropics and Colombia in particular, and a baseline for further study of distributional and biogeographic patterns of the suborder Auchenorrhyncha.

## Introduction

The natural history entomological collections represent a key and scientifically important sample of the world’s biological heritage and constitute the basis for fundamental and applied research (Suarez and Tsutsui 2004; Holovachov et al. 2014; National Academies of Sciences and Medicine 2020). To date, there are more than a million described insect species and more than half a billion preserved specimens, exceeding any other taxon (Short et al. 2018; Stork 2018). Some groups, such as hemipterans, lack detailed information and commonly are collected in biological expeditions but are not adequately characterized, classified, and preserved (Paradell and Defea 2017). They can be important specimens such as new species, extinct, endangered, cited, and/or historical material that should be identified and kept where they are safest or can most easily be studied (Llano et al. 2016). However, some taxonomic and societal preference biases are unavoidable (Troudet et al. 2017).

In megadiverse countries such as Colombia, the biological collections represent real bio- and databanks, natural heritage, and natural capital for current and further biodiversity studies (taxonomic, ecological, genetic, phylogenetic, and biogeographic), and are important elements for education, humankind’s knowledge, research, and conservation (Simmons and Muñoz-Saba 2005; Montaño Campaz et al. 2012; Ossa López et al. 2012; Trujillo-Trujillo et al. 2014; Arbeláez-Cortés et al. 2017). In Colombia, there are 286 biological collections registered in the Instituto Alexander von Humboldt (http://rnc.humboldt.org.co/admin/index.php/registros/colecciones (19 July 2022)) with that number tending to increase (Arbeláez-Cortés et al. 2017). Despite their importance, several concerns and challenges persist related to curatorial work, financial support, and lack of experts (taxonomists, para-taxonomists) in various taxonomic groups, among other concerns (Martinez Revelo and Medina Uribe 2017). This situation is much more marked for less charismatic insect species such as planthoppers and other hemipterans (Llano et al. 2016; Paradell and Defea 2017), where there is a clear underestimation of diversity (Forero 2008; Forero et al. 2018).

Issidae (Hemiptera: Auchenorrhyncha) is the fifth largest family in the infraorder Fulgoromorpha (Bartlett et al. 2014, 2018; Wang et al. 2016; Gnezdilov 2018a), with at least 217 genera and 1089 species (Gnezdilov 2018b, c; Gnezdilov et al. 2020; Bourgoin 2022). Recent work on the phylogenetic systematics of Issidae (Wang et al. 2016; Gnezdilov et al. 2020, 2022; Yang et al. 2021) have been informative on the evolution of Issidae, but are not in concurrence. New World Issidae, represented by Thioniini (which includes most New World taxa), is usually placed near the base of the tree, with *Picumna* Stål, 1864, excluded because of long-branch artifacts and the New World endemic tribes Guianaphrynini and Cordelini (Gnezdilov 2018a, 2019) excluded because of the absence of molecular data.

Both nymphs and adult issids are often associated with forested strata and many species are reported to be monophagous, with 50% of nymph and 64% of adult plant records from a single plant species (Wheeler and Hoebeke 1982; Wheeler and Wilson 1987; Wilson et al. 1994). Other species are described as “insects of economic importance” such as *Dentatissus damnosus* (Chou and Lu, 1985) attacking fruit-trees in China (Yan et al. 2005), *Agalmatium flavescens* (Oliver, 1791) affecting olive trees in Turkey (Lodos and Kalkandelen 1981), *Chimetopon camerunensis* Schmidt, 1910 on Cacao in western Africa (Gnezdilov 2013), among others. Additionally, some species are invasive, such as *Aplos simplex* (Germar, 1830) (as *Thionia*) in Europe (Gnezdilov and Poggi 2014; Gnezdilov 2018b), *Agalmatium bilobum* (Fieber, 1877) in California, USA (Gnezdilov and O’Brien 2006; Bartlett et al. 2014), and *Euroxenus vayssieresi* (Bonfils, Attié and Reynaud, 2001) in Hawaii, USA, and Ghana (Gnezdilov 2022; Gnezdilov and Bartlett 2022).

The genus *Thionia* Stål, 1859, is the largest New World genus of Issidae, including ~ 63 extant species (Gnezdilov 2009, 2013, 2018b, 2020; Bartlett et al. 2014, 2018; Bourgoin 2022), despite the recent transfer of species into new or revised genus concepts (Gnezdilov and Bartlett 2018; Gnezdilov and Dmitriev 2018; Gnezdilov et al. 2022). *Thionia* is the type genus of the Thioniinae Melichar, 1906 (sensu Wang et al. 2016; Thioniini sensu Gnezdilov et al. 2022). *Thionia* is widely distributed in the New World including North (Canada, USA), Central, and South America (Bartlett 2020; Bourgoin 2022). The generic diagnosis for *Thionia* Stål was recently refined by Gnezdilov (2018a), following the designation of a neotype for the type species *Issus longipennis* Spinola 1839 by Gnezdilov and Dmitriev (2018). Among American genera of Issidae, *Thionia* is recognized by possessing posterior tibiae with 2 (or 3) lateral spines and cubital vein of forewing simple (Bartlett et al. 2014). The genus is further characterized by having an “almost square” vertex bearing median and sublateral carinae anteriorly joined near carinate fastigium (fastigium approximately transverse in dorsal view); vertex and frons in lateral view joined at obtuse angle. Forewings longer than wide, rounded apically, exceeding abdominal apex; hindwings well developed, 3-lobed (Gnezdilov 2018a). *Thionia* lacks the elongate, tapered, downward projecting gonoplacs of *Cheiloceps* Uhler, 1895, a genus that appears limited to the Caribbean (Gnezdilov 2018a; Gnezdilov and Poggi 2014), although the diagnostic features of the gonoplacs for *Thionia* have not been elucidated.

The family Issidae Spinola, 1839 in Colombia is poorly known, with just nine species—two *Dracela* Signoret, 1861 (*D. annulipes* Signoret, 1861, *D. pehlkei* Schmidt, 1923) and seven *Thionia* Stål, 1859 (*T. colombiae* Walker, 1851, *T. dissimilis* Schmidt, 1910, *T. fusca* Melichar, 1906, *T. longipennis* (Spinola, 1839), *T. pehlkei* Schmidt, 1910, *T. prasina* (Spinola, 1839) and *T. proxima* Melichar, 1906)—reported in Colombia (Melichar 1906; Schmidt 1910, 1923; Metcalf 1958; Gnezdilov and O’Brien 2008; Bartlett 2020; Bourgoin 2022). This appears to greatly underestimate the real biodiversity of the group in Colombia (e.g., compared to Panama with 17 reported Issidae; Bourgoin 2022). This is a general situation for other Auchenorrhyncha and terrestrial hemipterans in Colombia, probably due to limited survey effort, lack of modern taxonomic tools, and few specialists in hemipteran taxonomists in the country (Llano et al. 2016).

Therefore, it is our interest to promote initiatives in support of Neotropical insect taxonomy and the current status of the genus *Thionia*, vouchered into certified insect collection resources of Colombia, as a pioneer information source for further insect biosystematic data, training, and research. This study begins to address the paucity of available distributional and ecological data for the country. Our main objective was to analyze available distributional data and host plant associations related to the genus *Thionia* Stål, 1859 in Colombia based on specimens examined from certified national biological collections, and relate these data to the ecoregions presented by Olson et al. (2001).

## Methods

Morphological nomenclature and taxonomy of the family follow Bartlett et al. (2014), Gnezdilov (2018a), and Gnezdilov and Dmitriev (2018). Specimens of Issidae were examined from the following Colombian collections:

- CEBUC: Colección de Entomología del Programa de Biología, Universidad de Caldas (Manizales, Caldas).
- EMPUJ-ENT: Colección de Entomología del Museo Javeriano de Historia Natural, Pontificia Universidad Javeriana (Bogotá, Cundinamarca).
- IAvH: Instituto Alexander von Humboldt (Villa de Leyva, Boyacá).
- ICN: Instituto de Ciencias Naturales, Universidad Nacional de Colombia (Bogotá, Cundinamarca).
- MEFLG: Museo de Entomología Francisco Luis Gallego, Universidad Nacional de Colombia (Medellín, Antioquia).
- UNAB: Colección Entomológica Universidad Nacional Agronomía Bogotá, Universidad Nacional de Colombia (Bogotá, Cundinamarca).
- UPTC: Colección Entomológica Museo de Historia Natural Luis Gonzalo Andrade, Universidad Pedagógica y Tecnológica de Colombia (Tunja, Boyacá).

Geographic information was recorded from the specimens’ labels (see Appendix 1). Distributional records were analyzed by matching collection records to ecoregions after Olson et al. (2001) using the freeware DIVA-GIS 7.5. Specimens of Issidae were not identified past genus because there are no modern taxonomic tools for Issidae of Colombia and primary types of relevant species are primarily in Europe. Legacy tools such as Melichar (1906) and original descriptions may have yielded speculative identifications, but we deemed these untrustworthy given the potential for many undescribed species as reported by Barringer et al. (2019) for Ecuador.

## Results

**Fig. 1:**
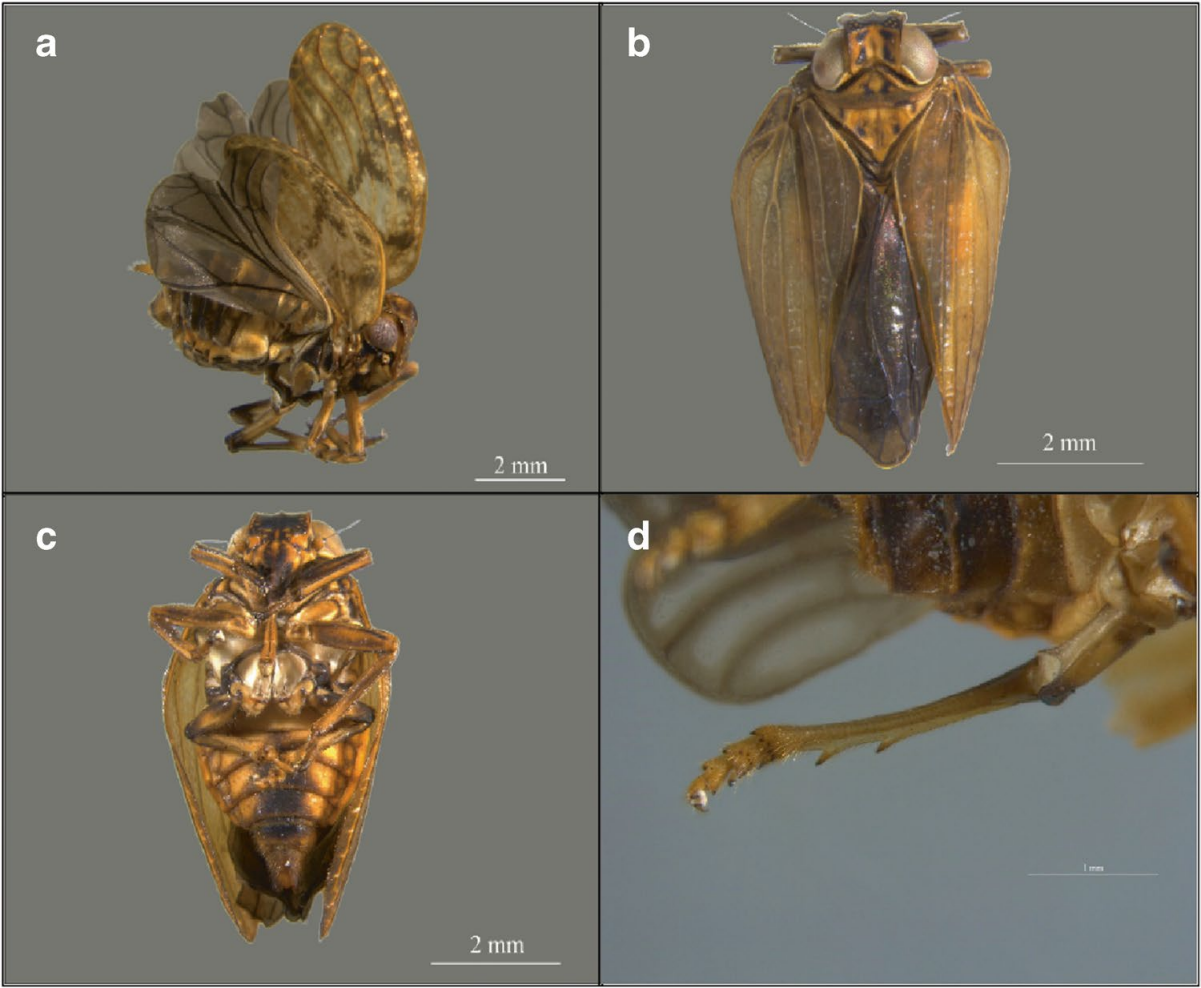
Colombian *Thionia* Stål, 1859. **a** Male lateral view, **b** Female dorsal view, **c** detail of posterior tibiae with 2 spines, **d** Female ventral view (photos by Camilo Llano)

**Fig. 2:**
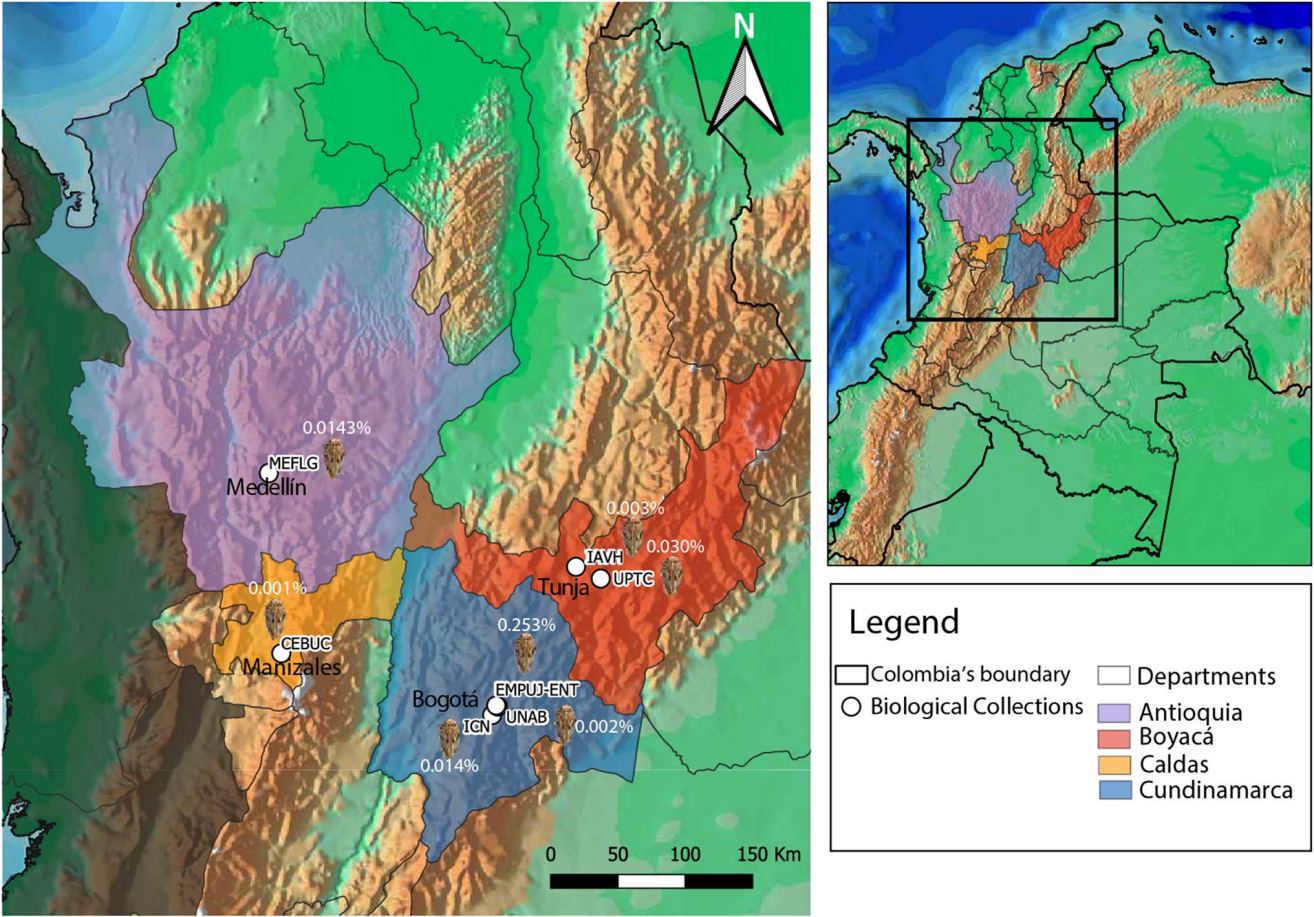
Percentage of representativeness of the different revised voucher specimens of *Thionia* Stål, 1859 in selected Colombian biological collection data sets (map by Mateo Rivera and Camilo Llano)

Our survey of Colombian natural history collections found 54 individuals of the genus *Thionia* Stål, 1859 (52 adults, 2 immatures [nymphs], Fig. 1). In all reviewed biological collections, the *Thionia* specimens represented less than 0.25% of the total arthropod voucher specimens (Fig. 2, Appendix 2). Metadata on collection methods found that collection by hand method was the most common (77% = 41 of records), malaise trap (19% = 10), sweep nets (2.8%), and pitfall trap (1.2%).

### Distributional and altitudinal records

**Fig. 3:**
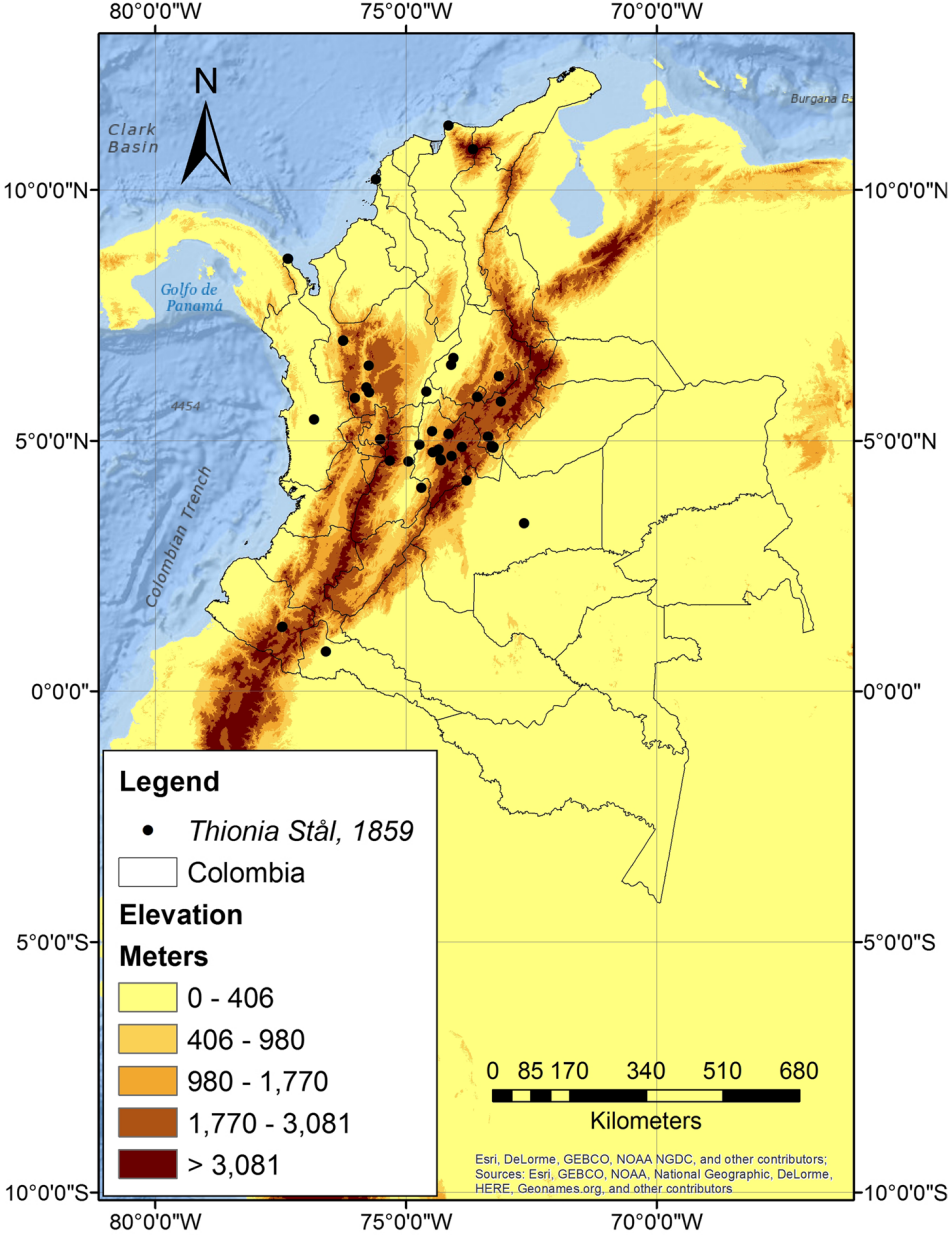
Distributional data for the genus *Thionia* Stål, 1859 in Colombia based on records from the certified biological collections; note that most of the records are associated with the Andean ecoregion (map by Jhon Faver Marulanda)

Altitudinal distributional records for Colombia range from 2 to 2550 m a.s.l. (Fig. 3). Our review revealed records associated with areas of biological importance or protected areas such as national natural parks, private ecological reserves, and/or botanical gardens (15 records; see Appendix 1). It also was apparent that *Thionia* was associated with forest fragments immersed in agroecosystems (8 records), which provide a refuge for these insects.

### Distribution of *Thionia* in the Colombian Ecoregions

**Fig. 4:**
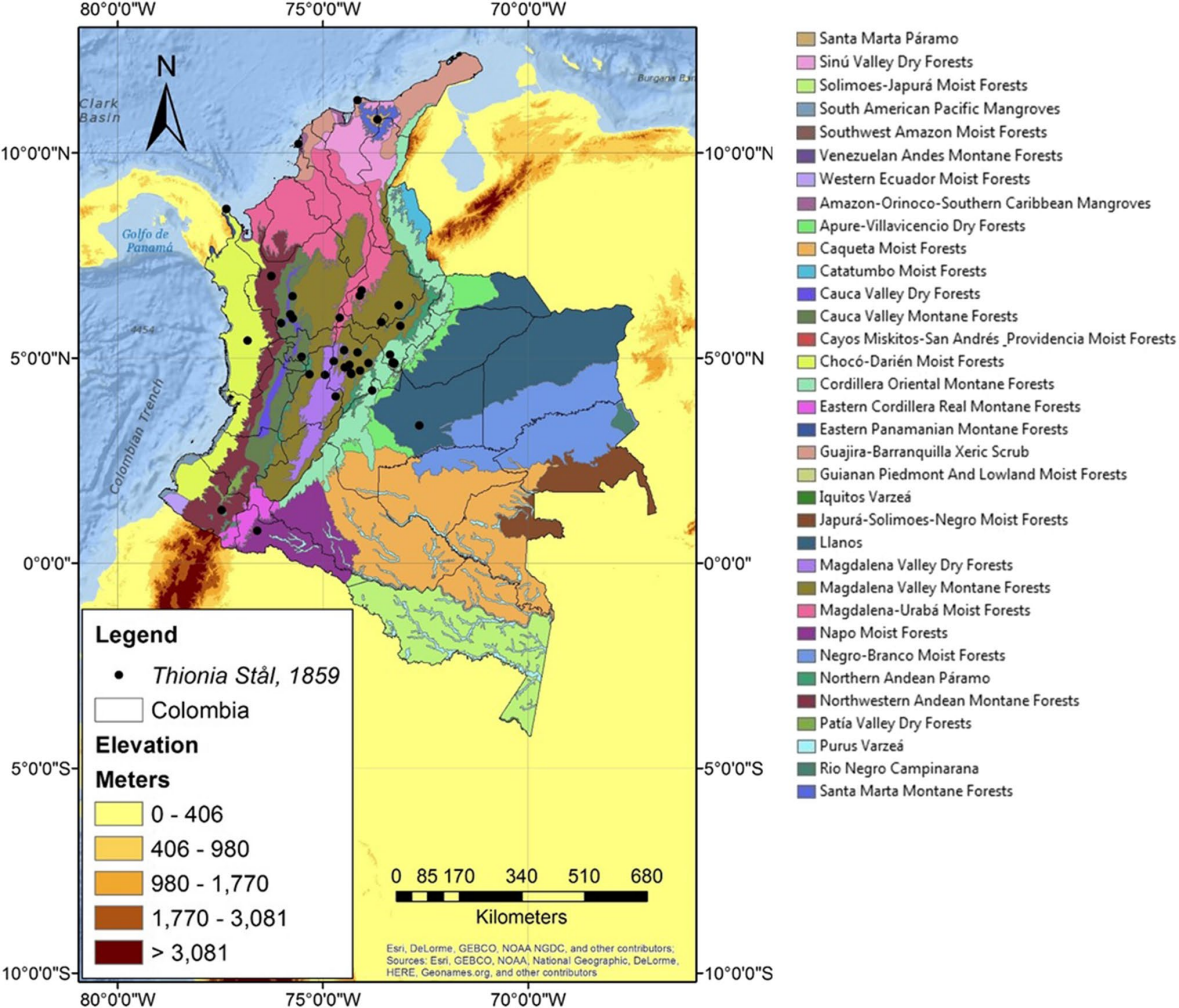
Distribution of *Thionia* records in Colombia in agreement with the ecoregions proposed by Olson et al. (2001) (map by Jhon Faver Marulanda)

Mapping specimen distributional data (see Appendix 1) on ecoregional areas proposed by Olson et al. (2001), showed most specimen records were in the Magdalena Valley Montane Forest. Other records were associated with the Santa Marta Westland, Cordillera Oriental, Magdalena Valley Dry Forest, Magdalena-Urabá Moist Forest, Cauca Valley Montane Forest, Cauca Valley Dry Forest, Chocó-Darién Mountain Forest, Northwestern Montane Forest, Purus Varzeá, Napo Moist Forest, and Llanos (Fig. 4).

## Discussion

Although reported collecting methods follow those expected (most hand collection), many records may be opportunistic. Colombian fulgoromorphs have been underrepresented in biological (terrestrial studies) or in studies on arthropods in general, as “occasional” finds and generally are scarce in collections (Osborn 1938), despite reports that Malaise and black-light traps can be useful tools for capturing Auchenorrhyncha from the canopy (Weber and Wilson 1981; Wilson 2005; Barringer and Bartlett 2018). Barringer et al. (2019) reported a large abundance and diversity of issids from forest canopy fogging samples in Ecuador, suggesting this to be an effective collecting method for issids.

### Distributional and altitudinal records

*Thionia* is a genus of mostly Neotropical distribution (Wheeler and Wilson 1988; Gnezdilov 2018b), reported from México, Honduras, El Salvador, Guatemala, Costa Rica, Panamá, Colombia, Venezuela, Ecuador, Perú, Bolivia, Guyana, French Guiana, Nicaragua, and Brazil (Bourgoin 2022), although some *Thionia* species extend into temperate North America (Bartlett et al. 2014). For Colombia, the genus is recorded for the first time at the departments of Boyacá, Magdalena, Meta, Chocó, Santander, Tolima, Bolívar, Caldas, and Antioquia. Early publications of Colombian records appear to mostly refer to the Cundinamarca department (see Fig. 2).

This distribution may be associated with the relationship between Issidae and their host plants due to the spatial distribution of plants jointly with their complexity and leaf architecture may limit the presence, richness, and abundance of Issidae and other planthoppers (Denno 1994; Wilson et al. 1994). In addition, it has been observed that fragmentation associated with the creation of agroecosystems changes the herbivorous community and multitrophic interactions with significant effects on the presence and distribution of selected taxa (Kareiva 1987; Gardarin et al. 2018).

### Distribution of *Thionia* in the Colombian Ecoregions

The presence of Issidae in Colombia appears to be significantly correlated to climate (e.g., precipitation and temperature) and biological metrics (e.g., vegetation structure) (Holdridge 1967; De Laubenfels 1975; Schmidthüsen 1976; Walter and Box 1976; Olson et al. 2001; Bailey 2014). Likewise, ecoregions may represent areas of marked endemism and promote conservation plans at the species or community level (Noss 1994; Olson et al. 2001; Dinerstein et al. 2017). Additionally, the Magdalena Valley Montane Forest, Cordillera Oriental, Magdalena Valley Dry Forest, Magdalena-Urabá Moist Forest, Cauca Valley Dry Forest, Chocó-Darién Mountain Forest, Purus Varzeá, and Napo Moist Forest are transformed ecosystems (Etter et al. 2020) due to anthropogenic activities as deforestation, ampliation of the agriculture, and livestock frontier (Armenteras et al. 2003; González et al. 2011). The Santa Marta Westland is categorized as Vulnerable (VU), Northwestern Montane Forest (VU), Cauca Valley Montane Forest, and Colombian Llanos as of Least Concern (LC) (Etter et al. 2020).

National biological collection faunistic records can be associated with regions currently under heavy human pressure using the IUCN Red List of Ecosystems and future risks (Bland et al. 2019; Etter et al. 2020). Despite the opportunity, the genus-level determination of *Thionia* species may mask the vulnerability of these issids to land use and land cover changes. Moreover, the ecological importance of Auchenorrhyncha and other Colombian terrestrial and riparian hemipterans is practically unknown (Llano et al. 2016), and this makes it difficult to promote conservation programs and alternative tools to manage pests or exotic insects in agriculture areas.

An issue to consider in further analyses is that these records are associated with a higher concentration of researchers, experts, research centers, and universities mainly in the Andean region of Colombia (other issues highlighted by Arbeláez-Cortés 2013 and Arbeláez-Cortés et al. 2017), which may geographically bias these data and it should be interpreted accordingly. Likewise, despite insects being highly studied in Colombia (Arbeláez-Cortés 2013), their classification at the lowest practical taxonomical level is heterogeneous, with some taxa identified only to family or supra-family level, and others undetermined even to these levels. This is related to the lack of taxonomic experts, as is the case of Hemiptera. In addition, the highlighted biological collections represent those with higher housing history and much better systematics curatorial background, specifically related to the genus *Thionia* (Fig. 2).

### Host plant associations

Plant associations previously reported for *Thionia* species include *Thionia borinqueta* Caldwell, 1951 on *Coccoloba uvifera* (L.) L. (seagrape, Polygonaceae); *Thionia bullata* (Say) on three pine species (Pinaceae): *Pinus taeda* L (Loblolly pine), *P. echinata* Mill. (Shortleaf pine), and *P. virginiana* Mill. (Virginia pine); and *Thionia elliptica* (Germar, 1830) on *Quercus ilicifolia* Wangenh. (bear oak) and *Q. marilandica* Münchh. (blackjack oak, Fagaceae) (Wheeler and Wilson 1987; Wilson et al. 1994; Palmer and Pullen 2001). Our review includes a plant association with *Theobroma cacao* L. (the cocoa tree, Malvaceae), a species of great commercial value in central-west Colombia (FEDECACAO 2019). Nonetheless, Issidae are seldom reported as pests and have not been demonstrated to be plant-pathogen vectors (Bartlett et al. 2018). In addition, found a “new” habitat association with a *Thionia* was reported from the mangrove forests of north-east Colombia (Atlántico department, Barú Island) where four mangrove species are present: *Rhizophora mangle* L., *Avicennia germinans* (L.) L., *Laguncularia racemosa* (L.) C.F. Gaertn., and *Conocarpus erectus* L. (Valle et al. 2011).

### Implications for conservation

As highlighted by Gnezdilov and O’Brien (2008) and recently by Gnezdilov and Bartlett (2018) and Gnezdilov (2019), contributions to the study of Central and South American issids have been made by few authors from the 1900s. A further purpose of our work is to contribute to the knowledge of Neotropical fulgoromorphs so that revision or description of the Colombian species can occur within an established ecological framework. In this sense, our review represents an advance in the knowledge of Issidae (and *Thionia* specifically) in Colombia and a baseline for further study of distributional and biogeographic patterns. However, only data from the biological collection were considered and we highly recommend sampling-based fieldwork (including in Colombian ecoregions where Issidae are not yet detected) for advancing ecosystem patterns and plant associations. In addition, taxonomic and systematics studies are necessary for establishing the current or potential distributions at the species level. Despite considerable progress in taxonomic studies, no phylogenetic treatment of the group had been published until recently (Wang et al. 2016; Gnezdilov et al. 2020, 2022). While we provide plant associations for *Thionia*, further research is needed to confirm their true host plant affinities. Although host preference varies among planthopper families, some being inhabitants of grass, herbaceous, and small riparian plants, others can be expected to occur in the forest canopy. However, it is often poorly documented (Barringer et al. 2019). Even though taxonomic and systematics requirements about insects are recognized worldwide, these represent more requested issues in megadiverse countries; similarly, as occurs with many arthropods, planthoppers should include several undescribed species in the tropics (Barringer et al. 2019). It has been apparent that the rich Neotropical issid fauna is still in its initial stage of discovery (Gnezdilov et al. 2020). Additionally, knowledge of their current and future entomological biodiversity due to natural and anthropogenic impacts is still largely unknown.

## Appendix

### Appendix 1

Examined material from Colombian departments and information extracted from the representative national collections (check collection abbreviation afore):

- **Antioquia:** 2 ♀♀ Colombia. Antioquia, Sandoná/La Joya. 10-V-1970. Col: Caicedo, F.; Benavides, H. [MEFLG]; 1♀ Colombia. Antioquia, Puerto Araujo. 0-IX-1945. Col: Gallego, F. [MEFLG]; 1♂ Colombia. Antioquia, Venecia. 1-V-1947. Malaise trap. Upon vegetation. Col: Gallego, F. [MEFLG]; 1♀ Colombia. Antioquia, Bolívar. 1-VII-1942 on *Theobroma cacao.* Col: Gallego, F. [MEFLG]; 1♀ 1♂ Colombia. Antioquia, Titiribí. 1-VI-1972 upon vegetation. Col: Madrigal, A. [MEFLG]; 1♀ Colombia. Antioquia, Sopetrán. 1-IX-1951. Col: Gallego, F. [MEFLG]; 2 ♀♀ Colombia. Antioquia, Dabeiba. 0-I-1945. Col: Gallego, F. [MEFLG].
- **Bolívar:** 1♀ Colombia. Bolívar, Cartagena, Isla Barú. 15-X-2006 on mangrove swamp forest. Altitude: 2 m. Col: Dias et al. [EMPUJ-ENT].
- **Boyacá:** 1♀ Colombia. Boyacá, Moniquirá. 8-VI-2004. Col: Arias, S. [UPTC]; 1♀ Colombia. Boyacá, Paipa. 1-VII-1974. Col: Avellaneda, M. [ICN]; 1♂ Colombia. Boyacá, Puerto Boyacá, Hacienda Aguaclara. 20-III-1970. Altitude: 600 m. Col: Arévalo, I. [ICN]; 1♂ Colombia. Boyacá, Puerto Boyacá. 11-III-1997. Altitude: 320 m. Col: Amat, G. [ICN]; 2♀♀ Colombia. Boyacá, Santa María, Sector La Almenara. 18-XI-2015. Hand collection. Coordinates: 4.87486 N–73.25508 W. Altitude: 1123 m. Col: Perdomo, M.; Torres, M. [EMPUJ-ENT]; 2♂♂ Colombia. Boyacá, Santa María. Edge forest/Sendero Hyca Quye. 18-XI-2015. Hand collection. Coordinates: 4.89811 N–73.29344 W. Altitude: 900 m. Col: Morales, J.; Cruz, J; Piña, M. & Pérez, C. [EMPUJ-ENT]; 1♀ Colombia. Boyacá, Santa María. Trail La Almería. Edge forest/upon vegetation. 17-III-2016. Hand collection. Coordinates: 4.87486 N–73.25508 W. Altitude: 1123 m. Col: Erazo, P.; Lesmes, C.; López, L. [EMPUJ-ENT]; 1♀ Colombia. Boyacá, Garagoa, Reserva El Secreto. 24-X-2001. Hand collection. Altitude: 2250 m. Col: Palacios, E. [EMPUJ-ENT]; 1♀ Colombia. Boyacá, Garagoa, Reserva El Secreto. 24-X-2001. Pitfall trap. Altitude: 2300 m. [EMPUJ-ENT]; 1♂ Colombia. Boyacá, Moniquirá, Vereda San Cristóbal. Hacienda La Loma. 10-X-2009. Hand collection. Altitude: 1700 m. Col: Piza, J. [UNAB].
- **Caldas:** 1♀ Colombia. Caldas. Villamaría, La Floresta. Agricultural matrix. On edge forest. Malaise trap. 28-VII-2015. Coordinates: 5°1′42.1″N–75°31′10.9″W. Altitude: 1814 m. Col: Llano, C. [CEBUC]; 2♀ Colombia. Caldas. Villamaría, La Floresta. Agricultural matrix. On edge forest. Malaise trap. 10-VI-2015. Coordinates: 5°1′42.1″N–75°31′10.9″W. Altitude: 1814 m. Col: Llano, C. [CEBUC]; 1♀ Colombia. Caldas. Villamaría, La Floresta. Agricultural matrix. On edge forest. Malaise trap. 18-XI-2015. Coordinates: 5°1′42.1″N–75°31′10.9″W. Altitude: 1814 m. Col: Llano, C. [CEBUC]; 1♂ Colombia. Caldas. Villamaría, La Floresta. Agricultural matrix. On edge forest. Malaise trap. 24-VI-2015. Coordinates: 5°1′42.1″N–75°31′10.9″W. Altitude: 1814 m. Col: Llano, C. [CEBUC].
- **Chocó:** 1♀ Colombia. Chocó. 8-IV-1978. Col: Echeverri, H. [ICN]; 1♂ Colombia. Chocó, Capurganá. Botanical garden. 23-IV-2007. Col: Chavez, P. [EMPUJ-ENT]; 1♀ Colombia. Chocó, Capurganá, Acandí. Forest. 8-X-2007. Hand collection. Altitude: 320 m. Col: Gonzales, D. [EMPUJ-ENT]; 1♀ Colombia. Chocó, Capurganá, Acandí. Forest. 8-X-2007. Hand collection. Altitude: 250 m. Col: García, S. [EMPUJ-ENT].
- **Cundinamarca:** 1♀ Colombia. Cundinamarca, Guasca. 7-I-1941. Altitude: 2400 m. Col: Richter, L. [ICN]; 1♂ Colombia. Cundinamarca, Anolaima. 18-VIII-1984. Col: Padilla, D. [ICN]; 1♀ Colombia. Cundinamarca, Chirajara. On the railway. 3-VI-1984. Col: García, M. [ICN]; 1♀ Colombia. Cundinamarca, Pacho, La Palma. 10-IX-1968. Col: Restrepo, M. [ICN]; 1♂ Colombia. Cundinamarca, El Colegio. Premountain wet forest. 1-VI-1984. Altitude: 990 m. Col: García, M. [ICN]; 1♂ Colombia. Cundinamarca, Bogotá D.C. 1-I-1942. Col: Richter, L [ICN]; 1♂ Colombia. Cundinamarca, Utica, La Palma. 27-X-1977. Col: Arévalo, L. [ICN]; 1♀ Colombia. Cundinamarca, Chicaque, Reserva Chicaque. Camping zone. 28-II-2014. Coordinates: 4.607950 N–74.30 W. Altitude: 2550 m. Col: Forero, D. [EMPUJ-ENT]; 1♀ Colombia. Cundinamarca, Chicaque, Reserva Chicaque. 12-IV-2013. Coordinates: 4.38673 N–74.18695 W. Altitude: 2540 m. Col: Figueroa, D. [EMPUJ-ENT]; 1♀. Colombia. Cundinamarca, Chicaque, Reserva Chicaque. On oak. 12-IV-2013. Coordinates: 4°37′0.35″N–74°18′37″W. Altitude: 2550 m. Col: Duque, N. [EMPUJ-ENT]; 2 nymphs. Colombia. Cundinamarca, Chicaque, Reserva Chicaque. 12-IV-2013. Coordinates: 4°37′0.35″N–74°18′37″W. Altitude: 2550 m. Col: Duque, N. [EMPUJ-ENT].
- **Magdalena:** 1♀ Colombia. Magdalena, Parque Nacional Natural Sierra Nevada de Santa Marta, Bella Vista. 15-V-2001. Malaise trap. Coordinates: 4.25472 N–73.39 W. Altitude: 1550 m. Col: Cantillo, J. [IAvH]; 1♂ Colombia. Magdalena, Parque Nacional Natural Sierra Nevada de Santa Marta, Bella Vista. 15-V-2001. Malaise trap. Coordinates: 4.25472 N–73.39 W. Altitude: 1550 m. Col: Cantillo, J. [IAvH]; 1♀ Colombia. Magdalena, Parque Nacional Natural Tayrona. 17-IX-1997. Heavy Duty Sweep Nets. Col: Kugler, C. [IAvH].
- **Meta:** 1♀ Colombia. Meta. Parque Nacional Natural Sierra de la Macarena, Caño Curia. 30-III-2003. Coordinates: 3.21 N–72.38 W. Malaise trap. Altitude: 150 m. Col: Villalba, W. [IAvH].
- **Putumayo:** 1♀ Colombia. Putumayo, Villa Garzón. Farm El Escondite. 19-IX-2015. Hand collection. Coordinates: 0°47′43″N–76°36′8″W. Altitude: 317 m. Col: Ariza, T. [IAvH].
- **Santander:** 1♀ Colombia. Santander, Puerto Parra, Campo Capote. 10-III-2008. Malaise trap. [ICN]; 1♀ Colombia. Santander, Charalá. 27-X-1978. Altitude: 1765 m. Col: Arévalo, L. [ICN].
- **Tolima:** 1♂ Colombia. Tolima. El Silencio. 2-VII-1986. Col: Sarmiento, L. [ICN]; 1♂ Colombia. Tolima. Cambao, Cerros de Santo Tomás. 12-XI-2000. Altitude: 250 m [EMPUJ-ENT]; 1♀ Colombia. Tolima. Cunday, Vereda El Edén. 1-I-1999. Altitude: 2450 m. Col: Morales et al. [EMPUJ-ENT].

### Appendix 2

Representativeness of the genus *Thionia* in arthropod’ voucher specimens from Colombian biological certified collections reviewed in the present study (source main: https://ipt.biodiversidad.co/sib/?request_locale=en (filtered by collection acronym))

**Table:**

Collection acronym	Total voucher specimen records	Total *Thionia* Stål, 1859	Percentage in collection (%)	Source
CEBUC	437,000	5	1.1441648 × 10^−3^	https://doi.org/10.15472/iify26
EMPUJ-ENT	7098	18	0.254	https://doi.org/10.15472/xmukx8
IAvH	116,170	4	3.44323 × 10^−3^	http://i2d.humboldt.org.co/ceiba/resource.do?r=colecicon_entomologica
ICN	100,000	14	0.014	https://doi.org/10.15472/vhqawn
MEFLG	6989	10	0.143	https://doi.org/10.15472/wf2h7t
UNAB	100,000	2	0.002	https://doi.org/10.15472/3jyev8
UPTC	3320	1	0.03	https://doi.org/10.15472/a0ugui
